# Mechanism of phase condensation for chromosome architecture and function

**DOI:** 10.1038/s12276-024-01226-x

**Published:** 2024-04-25

**Authors:** Jeongveen Park, Jeong-Jun Kim, Je-Kyung Ryu

**Affiliations:** 1 Department of Physics and Astronomy, Seoul National University, Seoul, 08826 South Korea; 2 Institute of Applied Physics of Seoul National University, Seoul, 08826 South Korea; 3 Institute of Molecular Biology and Genetics, Seoul National University, Seoul, 08826 South Korea; 4 Department of Biological Sciences, Seoul National University, Seoul, 08826 South Korea; 5 Interdisciplinary Program in Neuroscience, Seoul National University, Seoul, 08826 South Korea

**Keywords:** Chromatin structure, Single-molecule biophysics

## Abstract

Chromosomal phase separation is involved in a broad spectrum of chromosome organization and functional processes. Nonetheless, the intricacy of this process has left its molecular mechanism unclear. Here, we introduce the principles governing phase separation and its connections to physiological roles in this context. Our primary focus is contrasting two phase separation mechanisms: self-association-induced phase separation (SIPS) and bridging-induced phase separation (BIPS). We provide a comprehensive discussion of the distinct features characterizing these mechanisms and offer illustrative examples that suggest their broad applicability. With a detailed understanding of these mechanisms, we explore their associations with nucleosomes and chromosomal biological functions. This comprehensive review contributes to the exploration of uncharted territory in the intricate interplay between chromosome architecture and function.

## Chromosomal phase separation: unraveling mechanisms and physiological implications

Chromosomal phase separation, a process where a mixture splits into two separate parts, is important for various chromosomal functions (activities of chromosomes, the part of a cell that carries genetic information). However, understanding this process requires knowledge of polymer physics (the study of large, complex molecules). This study by Ryu and colleagues explores the patterns behind the molecular mechanisms (processes at a microscopic level) of chromosomal phase separations. The researchers reviewed the role of proteins and DNA molecules in this process. They discovered two distinct working models, Bridging-Induced Phase Separation (BIPS) and Self-association Induced Phase Separation (SIPS), are involved. The study concludes that the mechanism of chromosomal phase separation relies on the dynamics of proteins. This research enhances our understanding of chromosomal functions and could impact future studies in biological sciences.

This summary was initially drafted using artificial intelligence, then revised and fact-checked by the author.

## Introduction

Chromosomal phase separation has been found to be involved in various chromosomal functions, such as the formation of nuclear membraneless organelles^1–3^, heterochromatin, and transcriptional condensates^4,5^. Phase separation refers to the physical segregation of a single homogeneous mixture into two distinct phases^6^. Many biological questions and intracellular phenomena have recently been interpreted through the phase separation scheme^7–13^ because phase separation can explain long-standing biological questions such as the formation of membraneless organelles^14–18^, chromatin organization^19–22^, and signaling^23–26^, which cannot be explained by previously known structure‒function relationships. Phase separation is critical for organizing chromosome structure and managing chromosomal functions. However, because of the interactions between proteins and extremely long DNA molecules, understanding chromosomal phase separation requires polymer physics. In this review, we introduce patterns underlying the molecular mechanisms of chromosomal phase separation and present various examples. In addition, we extensively discuss physiologically relevant working models by factoring nucleosomes into these mechanisms and how the mechanisms relate to the function of phase-separated condensates.

## Chromosomal phase separation

**Fig. 1:**
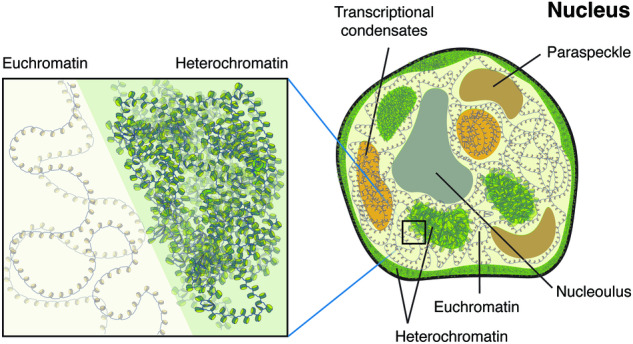
Condensates and chromatin in the nucleus: nucleolus, paraspeckles, transcriptional condensates, euchromatin, and heterochromatin. Heterochromatin covers the edges of the nucleus, and transcriptional condensates contain euchromatin for transcription. The inset shows an enlarged view of the boundary between euchromatin and heterochromatin regions with loosened and compacted chromatin structures, respectively.

The relationship between chromosome architecture and function is closely linked to a myriad of biological processes. First, chromosome structuring, or the packaging of extremely long DNA molecules into micrometer-scale structures, is achieved through phase separation^27^. Second, mitotic/meiotic chromosome structuring is used to deliver identical amounts of genomic information to daughter cells. Third, gene expression is regulated by chromosome structure. For example, heterochromatin has a closed chromatin structure that is tightly compacted to silence gene expression, while euchromatin has an open chromatin structure in which gene expression is highly activated. Finally, functional membraneless organelles in the nucleus (Fig. 1), such as the nucleolus^3,28,29^, paraspeckles^30,31^, transcriptional condensates^32–34^, and even X chromosome inactivation^35,36^, affect chromosome structure. Therefore, understanding chromosome architecture is key to understanding chromosomal function.

The key commonality of chromosomal phase separation is that extremely long DNA molecules are involved in forming condensates and that the biomolecules undergoing phase separation are extreme heteropolymers consisting of myriads of different combinations of DNA sequences and DNA-interacting proteins. Hence, polymer physics is needed to understand the phase separation process in chromosome organization and function^37^. In this unique environment, not only protein‒protein interactions but also protein‒DNA interactions should be considered to understand the molecular mechanism of chromosomal phase separation. DNA topology, such as loops, is typically involved in chromosomal phase separation. Thus, the molecular grammar of chromosomal phase separation might be different from the molecular grammar of non-chromosomal phase separation^38,39^.

## BIPS and SIPS

Two distinct working models for chromosomal phase separation have been suggested based on distinct polymer models (Fig. 1 and Table 1)^38,40–42^. The first working model is bridging-induced phase separation (BIPS, also known as polymer–polymer phase separation^21^), which uses multivalent protein‒DNA interactions instead of multivalent protein‒protein interactions. Multivalent protein‒DNA interactions can bridge two distinct DNA regions and form a DNA loop that acts as a nucleation structure for phase condensation. Another working model is self-association-induced phase separation (SIPS), in which multivalent protein‒protein interactions organize a protein assembly that interacts with DNA to form DNA/protein clusters.

**Table 1:** Criteria for BIPS versus SIPS^38^.

Criteria	BIPS	SIPS
Biochemical features of protein	Multivalent protein‒DNA interaction, IDR not necessary	Multivalent-protein‒protein interaction mainly by IDR^38^
Driving force	DNA bridging-induced attraction	Self-associated protein attraction
Nucleation process	The DNA-bridging region functions as a nucleation point	N/A
Growth process	Multivalent DNA-binding proteins accumulate at the DNA-bridged site until binding sites on chromatin are saturated with proteins.	Ostwald ripening^46^
DNA length-dependent behavior	Power-law scaling behavior (>3 kbp), no cluster formation on shorter DNA (<3 kbp)^40^	No specific length-independent behavior
Concentration influence	Cluster density^21^	Cluster size^21^

**Fig. 2:**
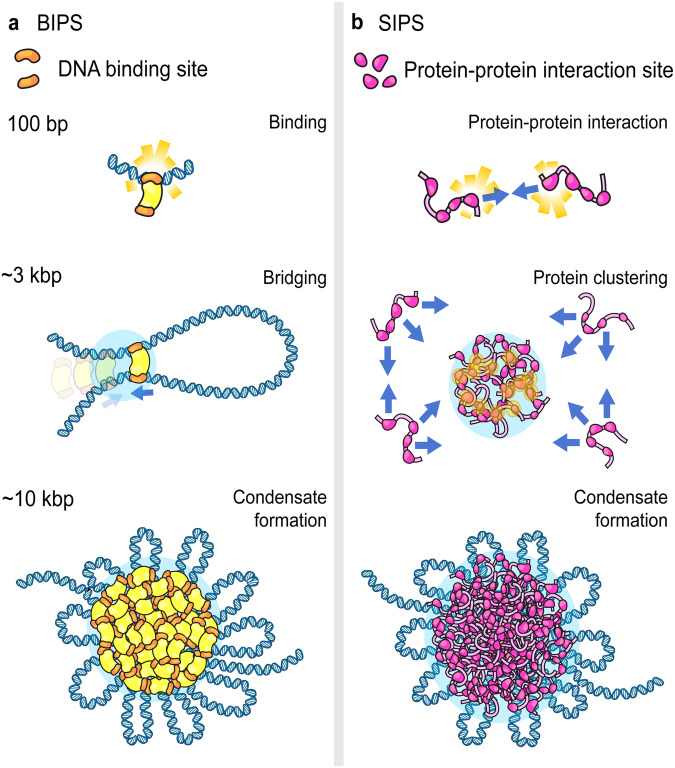
Schematic drawing of the molecular mechanism of BIPS versus SIPS. **a** SIPS is driven by multivalent protein‒protein interaction sites, normally in IDRs. A multivalent protein‒protein binding site (pink) in each protein can induce protein cluster formation, and the clusters interact with DNA to organize chromatin structure. **b** BIPS shows DNA length-dependent protein binding (~100 bp), DNA‒protein-DNA bridging (~3 kbp), and DNA‒protein clustering (~10 kbp). A protein with multivalent DNA‒protein binding sites (orange) can bind and bridge DNA to induce phase condensation.

We discuss the key features of BIPS and SIPS and the differences between these two mechanisms (Table 1). The mechanism is determined by the biochemical features of phase separating proteins. The protein that induces BIPS has multivalent DNA-binding sites that can induce DNA‒protein-DNA bridging to form a DNA loop as a nucleation point for further phase separation (Fig. 2a). In contrast, the protein that induces SIPS has multivalent protein‒protein interaction sites (Fig. 2b). Therefore, BIPS is strongly dependent on DNA-binding affinity for the nucleation of phase separation, whereas DNA‒protein interactions are not necessary for SIPS. SIPS is typically a chromatin-independent phase separation process that occurs not only in chromatin phase separation but also in other phase-separated bodies that are not involved in chromosomal biological processes. However, BIPS is strongly dependent on DNA‒protein interactions, and hence, it is specifically involved in chromosomal phase separation. Normally, intrinsically disordered regions (IDRs) are involved in multivalent protein‒protein interactions. This interaction can be explained by a stickers-and-spacers framework, where stickers are the protein‒protein interaction regions, and spacers are the noninteracting regions between the interaction sites^43^. IDRs contain multiple sticker regions, and their flexibility allows geometrically unconstrained interactions between proteins, promoting self-interaction. IDR is a key factor that triggers SIPS; however, in some proteins, such as CTCF, the presence of an IDR is not necessary for self-interaction and phase separation^44^. Another class of phase separation proteins similarly explained by the stickers-and-spacers framework is proteins with tandem interacting folded domains, such as the poly-SIM or poly-SUMO systems^45^. However, neither IDRs nor tandem domains are necessary for BIPS because DNA already presents diverse configurations and multiple chromatin-bridging sites where proteins can cluster.

These key differences in proteins determine the differences in the nucleation and growth of chromosomal phase separation. The nucleation of BIPS occurs at a bridged DNA region, and multivalent DNA-bridging proteins accumulate on the bridged region in the growth phase to complete phase separation. In SIPS, protein‒protein interactions serve as a nucleation point that provides multiple protein binding sites to trigger the transition of protein binding to the growth phase. In BIPS, once chromatin is fully bound by proteins, no additional growth can be observed, whereas the growth limit of SIPS is limited by Ostwald ripening, which is determined by the competition between the kinetics of protein‒protein interactions and the diffusion of each phase-separated droplet^46^. A recent study on a pioneer transcription factor revealed a switch-like transition from a thin adsorbed layer to a thick condensed layer, indicating a prewetting transition^47^. This illustrates an additional nucleation mechanism for SIPS wherein protein‒DNA surface interactions act as nucleation points for phase condensation, and the protein layer on the surface recruits additional phase-separating proteins via multivalent protein‒protein interactions. Moreover, capillary forces between two distinct condensates on distinct DNA regions can lead to the growth of a condensate^48^.

Another key difference between the two mechanisms is that either the droplet size or the density of protein in the droplet changes with the bulk protein concentration. As the bulk protein concentration increases, in BIPS, the condensate, once formed, will increase in internal protein concentration as proteins occupy more binding sites on the DNA. In SIPS, the droplet will maintain density but grow in size because the protein‒protein interactions and distances are not dependent on the bulk concentration^21^.

## Criteria for distinguishing condensates induced by BIPS or SIPS

Phenomenologically, BIPS and SIPS seem to produce similar DNA/protein condensates, but the molecular mechanism by which a protein induces the condensates is different, introducing the need for criteria to distinguish the condensates induced by BIPS or SIPS. In particular, the nucleation process of BIPS is induced by DNA topology changes mediated by proteins. BIPS is dependent not only on the presence of DNA but also on the DNA length, which can be used to distinguish BIPS from general SIPS droplet formation (Fig. 2a)^38^. Importantly, this phase separation necessitates longer DNA (more than 3 kbp) to facilitate the formation of distinguishable condensates. This is driven by BIPS, which depends not only on multivalent DNA-binding proteins but also on the great length of DNA polymers.

When observing BIPS, the optimal range for the length of DNA is between 100 bp and 10 kb. DNA is rigid, with a persistence length of 50 nm^49^, and a minimum DNA length is necessary for bending. The behavior of DNA-bridging proteins varies depending on the DNA length. A length of 100 bp results in binding. At several kbp, bridging occurs, and for DNA lengths of tens of kbp or more, condensates can form. The typical size of loops observed in freely fluctuating DNA is balanced between the energy required for bending and the entropy associated with looping. Therefore, the objective is to minimize the following free energy:

$$\frac{F}{{k}_{B}T}=\frac{2\varepsilon {l}_{p}}{l}+c\log \left(\frac{l}{{l}_{p}}\right),$$

where the first term is the bending energy of a generalized shape that is not strictly a perfect circle and can contain a kink. This freedom is described by the parameter $\varepsilon$. The second term is the entropic loss due to a loop of size $l$, computed as $-{k}_{B}T\log [{(l/{l}_{p})}^{-c}]$, where ${l}_{p}$ is the persistence length. The exponent $c$ characterizes the contact probability of two segments in a polymer (e.g., $c=1.5$ for an ideal random walk). Setting $\varepsilon =16$, which is valid for a teardrop shape^50^, we obtain that a minimum free energy of ${l}^{* }=3.2$ kbp.

This calculation suggested that DNA segments should be longer than 3 kbp to observe looping and bridging-induced clustering, although the specific threshold might differ with the details of each experiment. Clustering is entropically and energetically favored over dispersion because the proteins can bridge DNA on the existing loop without needing to form new loops, which would cost energy and entropy. This positive feedback, where looping attracts proteins that further drive looping, is called bridging-induced attraction^51,52^. Therefore, bridging causes the transition of the DNA into an ordered globular compartment^21^.

## Candidate proteins for the induction of BIPS and SIPS

In Table 2, we list some candidates for proteins that enable BIPS or SIPS nucleation, with candidates for BIPS identified on the basis of imaging data that show proteins bridging DNA. We will clarify the reasons for the identification of BIPS and SIPS and aim to interpret and introduce the latest research findings that support this claim. First, we explored the properties of phase-separating proteins based on whether the proteins induced phase separation via multivalent protein‒protein interactions or multivalent protein‒DNA interactions. To examine this criterion, we determined whether the protein exhibited phase separation at a physiologically relevant concentration in the absence of DNA. Another possible criterion is whether an IDR is involved in multivalent protein‒protein interactions, as IDRs contain multivalent protein‒protein interaction sites that can be described by a sticker-spacer model^43^.

**Table 2:** Candidate proteins in BIPS/SIPS.

Protein	Imaging method	Phase separation type	Driving interaction	Function	References
Cohesin	Single-molecule DNA tethered assay and AFM	BIPS	Multivalent DNA‒protein interaction	Interphase chromosome organization	^40^
ParB	Single-molecule DNA tethered assay	BIPS	Multivalent DNA‒protein interaction	Bacterial chromosomal organization for sporulation	^64,87^
H-NS	AFM	BIPS	Multivalent DNA‒protein interaction	Bacterial chromosomal organization for gene silencing	^79^
NPM1	FRAP	SIPS	IDR	Construction of nucleus as a site for ribosomal RNA transcription	^9^
FUS	SIM83	SIPS	IDR (residue 42~421)^74,115^	Retention of proteins and RNAs to constitute paraspeckles^47^	^74,83,115^
Pol II	PALM	SIPS (?)	IDR, etc.	Control of RNA transcription function in transcription condensates	^5^
Nopp140	FRET^47^	SIPS	IDR^116^	Maintenance of Cajal body by multivalent interactions with Coilin	^84,116^

## Examples of BIPS

### Cohesin

Cohesin plays a crucial role in chromosome organization at interphase as a member of the structural maintenance of chromosomes (SMC) family^53–55^. It has been proposed that the cohesin complex extrudes a DNA loop to organize the interphase chromosome structure and holds two sister chromatids before chromosomal segregation, and this complex might be used for the regulation of transcription, DNA replication, DNA repair, and more chromosomal activities^56^. Notably, it has recently been shown that the cohesin phase separates along DNA *via* BIPS^40^.

A previous study showed that the co-condensation of cohesin/DNA molecules depends on DNA–cohesin interactions. Moreover, BIPS was confirmed by a DNA length control experiment performed using an atomic force microscope (AFM). At DNA lengths less than 3 kbp, cohesin did not induce cluster formation, although a single cohesin was able to bind to a DNA molecule. However, above a DNA length of 3 kbp, cohesin induced cohesin/DNA cluster formation with a power-law behavior depending on the DNA length (the cluster size: $R\propto {l}^{\alpha }$, where *l* is the DNA length and is the power-law exponent), and the power-law behavior and the exponent, $\alpha$ = 0.45, agreed with BIPS due to the bioconnectivity of the cohesin complex to DNA. Furthermore, in silico Hi‒C maps from molecular dynamics (MD) simulations showed weaker compartmentalization by cohesin-mediated BIPS, although proteins that can bridge DNA at multiple points (≥10) were able to construct strong compartmentalization patterns. This result indicates that BIPS by cohesin can provide another building block for genome organization. However, it is still a mystery how DNA-loop extrusion and compartmentalization occur together in genome organization.

### Partition protein B, ParB

ParB is a protein involved in the ParAB*S* system that participates in bacterial chromosome segregation, specifically in the partitioning of plasmids and certain bacterial chromosomes during cell division^57–59^. Because ParB acts as the main motor to transfer genomes to differentiated cell sites, the main mechanism is driven by CTP hydrolysis^60–62^. In vivo and in vitro experiments showed that ParB induced phase separation ParB^62,63^. Specifically, in vitro phase separation of ParB was observed in an environment containing dsDNA or plasmid, regardless of the type of crowding agent used^62^. ParB contains multivalent DNA-binding sites, one of which is known to target the specific sequence *parS*^58^. Hence, ParB can bridge pairs of distinct DNA segments. Furthermore, ParB was shown in single-molecule experiments^64^ and computer simulations^65^ to spread along DNA to recruit additional ParB molecules to DNA for bridging. Therefore, ParB can be considered a BIPS candidate. Additionally, the cluster size of ParB-*parS* is independent of the ParB concentration^66^, which might be related to the size limit of microphase separation.

### Kruppel-like factor 4, Klf4

The zinc finger transcription factor Klf4 is a key constituent of reprogramming-induced pluripotent stem (iPS) cells^67,68^. Klf4 performs a DNA bridging function with three zinc fingers that bind to GC-rich regions of DNA to mediate the activation and repression of transcription^69–71^. The bridging of DNA by Klf4 zinc fingers has been observed by in vitro single-molecule fluorescence resonance energy transfer (smFRET)^72^. Additionally, Klf4 does not require an IDR for phase separation, whereas IDRs are considered to play a crucial role in SIPS^73–75^. On the other hand, the DNA binding domain (DBD) of Klf4 has been confirmed to be an essential factor in its formation of phase-separated droplets^72^. In the presence of longer DNA (7.4 kbp), Klf4 exhibits robust phase separation at notably lower concentrations (~ 250 nM), which strongly suggests an instrumental role of bridging mechanisms under physiological conditions. However, Klf4 condensation without DNA was observed only at concentrations higher than the physiological nuclear concentration (~1 µM)^47^. In addition, the Klf4 DBD phase separates in the presence of short DNA (30 bp) but at a nonphysiological concentration (~ 6 µM)^72^. These results suggest that SIPS by Klf4 occurs at high concentrations. This finding is consistent with a recent experiment that showed a prewetting transition along DNA stretched by dual-optical traps^47^. BIPS is more likely to occur at the initial stage when DNA is not fully stretched, whereas after DNA is stretched via BIPS by Klf4 and a large enough number of proteins are bound to the DNA, SIPS is more likely to occur by prewetting transition. This result suggested that Klf4 might participate in dual pathways depending on the protein concentration. However, how Klf4 behaves in living cells remains an open question.

### Histone-like nucleoid structuring protein, H-NS

The DNA bridging mechanism was first proposed to explain the behavior shown by the protein H-NS^76,77^, which interpreted by considering DNA as a polymer model and showing that compartmentalization is induced by bridging spatially close DNAs^78,79^. DNA bridging by H-NS was first observed through AFM, confirming the hypothesis that H-NS mediates DNA bridging^80^. Moreover, H-NS facilitates DNA bridging through dimerization^81–83^. In addition, a simulation study concluded that when H-NS undergoes dimerization, it forms moderately condensed DNA if cis-binding is stable, whereas if trans-binding is stable, it promotes the formation of a globule^79^. DNA bridging by H-NS involves nucleation at a promoter, followed by spreading and condensation^80^. This evidence suggests that H-NS is an example of a protein that organizes BIPS.

## Examples of SIPS

Some chromosomal phase-separating proteins do not have multivalent DNA interaction sites; instead, they form multivalent protein–protein interactions with their IDRs. Many examples can be found, and here, we list some representative candidates that form SIPS. Nucleophosmin (NPM1), a major component of the nucleolus granular component, initiates phase separation through its multivalent IDR and can form nucleolar condensate droplets for ribosome biogenesis^9^. Fused in sarcoma (FUS), a constituent of paraspeckles, has also been revealed to undergo substantial phase separation through multivalent interactions between IDRs^75^. In particular, FUS is located centrally, in contrast to Rbm14, which is localized near paraspeckles, making it a plausible candidate for condensation^84^. This nuclear body does not contain chromatin and does not require bridging but regulates chromosomal functioning by controlling the expression of specific genes through the retention of various proteins and RNAs. Transcription condensates comprise a variety of proteins, including the mediator complex and RNA polymerase II (Pol II). This accumulation into a liquid condensate has been reported by Cho et al.^5^. The mediator complex, a component of the transcription factory, is known to undergo phase separation via the IDR^32^. Nopp140 forms a condensate via its IDR and interacts with coilin, a component of the Cajal body^85^. Notably, the IDR of Nopp140 adheres to the N-terminal domain (NTD) of coilin, contributing to the formation of the Cajal body.

## Categories of BIPS in chromosomal organization

**Fig. 3:**
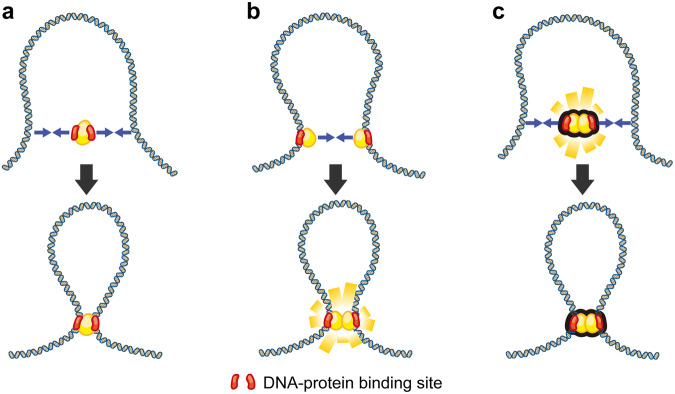
Distinct DNA bridging models in the BIPS nucleation process. **a** Bridging by a single protein (yellow) with multivalent DNA-binding sites (red). **b** Bridging interaction between DNA prebinding proteins with a single DNA-binding site. **c** DNA bridging by predimerized (or preoligomerized) proteins with a single DNA-binding site.

BIPS-inducing proteins can interact with DNA differently due to their dimerization and diffusivity. We classified BIPS into three types based on how DNA bridging is induced (Fig. 3). For instance, H-NS or ParB dimerizes and provides two DNA-binding sites per single-unit dimer, resulting in DNA-bridging capability. In comparison, cohesin does not require dimerization (or oligomerization) to bridge DNA, but the complex can bridge two distinct DNA segments. Hence, these biochemical features induce BIPS by different mechanisms. In addition, some chromosomal proteins can diffuse along DNA, which influences the bridging mechanism. One-dimensional diffusion along a DNA molecule enables single-unit capture of another DNA segment for bridging; alternatively, two diffusing proteins can dimerize, creating a bridged DNA loop. In the first model, a single-unit protein (or complex) has multiple DNA-binding sites and performs DNA bridging by itself (Fig. 3a). One example is cohesin, which exhibits multivalent DNA binding to a single unit complex, allowing it to bridge two distinct DNA regions and induce phase condensation^86^. Additionally, since Klf4 has multivalent DNA-binding sites in a single unit, it can be considered to belong to the same category. In the second model, each protein first proceeds along the DNA strand by diffusive movement and subsequently attaches to another protein to form a bridge (Fig. 3b). For example, ParB first binds with DNA in its dimer state and then bridges DNA by interacting with another prebound dimer^87^. The final model differs from the previous model in terms of the order of binding and bridging: the unit protein forms a dimer (or oligomer) to obtain multivalent DNA-binding sites as a unit complex and directly performs DNA bridging upon binding (Fig. 3c). For example, H-NS dimerizes before DNA binding and bridging^88^. Once DNA bridging is induced, condensate growth occurs by the same mechanism because, entropically, the bridging point is the primary target of additional bridging proteins.

**Fig. 4:**
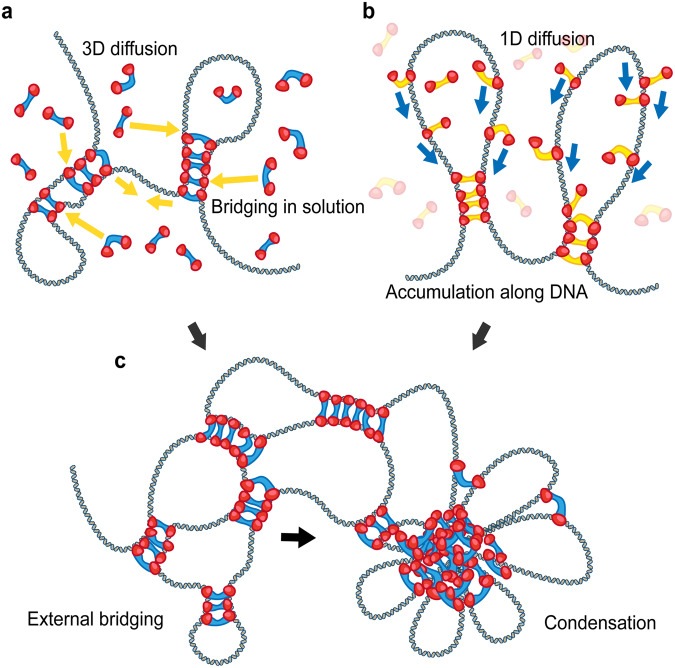
Diffusive bridging mechanism to form condensate. **a** DNA recruits proteins in solution via 3D diffusion to form bridging complexes. **b** Prebound proteins slide 1-dimensionally along a DNA molecule to form bridging complexes. **c** Bridged proteins aggregate DNA to form a condensate.

These different categories suggest that the mechanism of chromosomal phase separation is dependent on protein dynamics. Although proteins that cannot diffuse along DNA can bridge two distal DNA regions through the 3D diffusion motion of both DNA and proteins (Fig. 4a), proteins that can diffuse along DNA (1D diffusion) induce the accumulation of more proteins at the bridged region (Fig. 4b). These two different diffusive behaviors induce different mechanisms of the growth phase of condensation (Fig. 4c).

## Chromosomal phase separation in chromatin

For a more physiological description of chromosomal phase separation, we should consider how nucleosomes are involved. Nucleosomes can change the stiffness or bending of chromatin and provide additional binding sites for phase-separating proteins. The epigenetic modification of histones can also be involved in interactions between nucleosomes and phase-separating proteins. However, it is not yet clear how chromosomal phase separation occurs in chromatin in the presence of nucleosomes. We discuss how chromosomal phase-separating proteins interact with nucleosomes and the potential molecular mechanism of chromatin phase condensation.

We can categorize the types of chromatin interactions with phase-separating proteins into (1) multivalent bare-DNA-binding sites, (2) both bare-DNA binding sites and nucleosome binding sites, and (3) multivalent nucleosome binding sites. For example, proteins such as HP1α and polycomb repressor complex (PRC) are known to regulate chromatin compaction via epigenetic modifications. These proteins have exhibited different interaction modes depending on the experimental method, cell type, and developmental status. The key features are how these proteins interact with chromatin, and whether the imaging data or protein structure suggests BIPS (Table 3).

**Table 3:** Chromatin phase-separating proteins.

Protein	Species	SIPS/BIPS	Protein binding mode	Method	Phase	ETC	References
H1	Chicken	SIPS	Interaction with DNA and self-association through C-terminus IDR	In vitro	Liquid	Phosphorylation of C-terminus tail reduces interaction.	^117^
	Human			HeLa cell imaging	Liquid		^88^
HP1α/a	Human	SIPS	Hinge interaction with DNA, NTE (N-terminal extension) interaction with Hinge	Single-molecule DNA curtain assay	Liquid		^19^
	Drosophila			Drosophila, high-resolution 4D analysis using lattice light-sheet microscopy	Liquid (?)	The condensate is not entirely liquid and has static compartments.	^20^
	Human	BIPS	CD interaction with H3K9me3 mark on nucleosome	Cryo-EM			^90^
	Mouse			Confocal imaging of fibroblast chromocenter	Collapsed globule	The condensate has impermeable boundaries and exhibits concentration buffering, with coil-to-globule transition.	^118^
	Human			Hi-C		Heterochromatin-like structures can coalesce with constitutive heterochromatin.	^91^
	Drosophila			Confocal microscopy of transgenic *Drosophila* polytene		LacI-HP1a fusion protein induced bridging with distant chromosome sites on polytene.	^119^
	Drosophila	Both		Simulation & *Drosophila* embryo pericentromeric heterochromatin live-cell imaging	Liquid	Shows both characteristics of BIPS and SIPS. Condensate characteristics may differ with cell cycle and differentiation.	^92^
PRC1	Mouse	SIPS	LCDR (Low-complexity disordered region) of CBX2	FRAP with nucleosome controls	Liquid		^120^
	Mouse	BIPS (?) by histone bridging	AT hook of CBX2 interacts with DNA of chromatin	Live-cell single-molecule tracking (SMT, of mESCs)	Liquid	Condensate formation accelerates the target-search process. Eliminating the AT hook affects condensate formation considerably more than CD.	^33^
	Human		(RING Subunit) E3 Ubiquitin ligase RNF2 ubiquitinates H2AK119	Removal by RNA interference, ChIP			^98^
PRC2	Human	BIPS (?) by dimerization	The N-terminal C2 domain of SUZ12, one of the PRC2 subunits, interacts with the surface of RBAP48	X-ray crystallography			^121^
	Human			Cryo-EM		Dimers promote compaction	^122^
	Human	BIPS (?) by histone bridging	SET domain of EZH2 subunit interacts with the substrate histone’s nucleosome H3 tail.	Cryo-EM			^100^
	Drosophila		EED interacts with the H3K27me3 histone mark.	Crystallography and pull-down assay			^123^
	Human	BIPS (?) by multivalent DNA interaction	Various PRC2 subunits such as EZH2 SBD, CXD, (AEBP2), and EED interact with nucleosomal DNA	Cryo-EM			^100^
			Multivalent interaction with DNA	AFM		DNA is bent by monomers, bridged by dimers.	^124^

H1, the linker histone, undergoes phase separation with both DNA and nucleosomes^89^. H1 has a short flexible N-terminal tail, a central globular domain, and a long C-terminal IDR. The cryo-EM structure of H1 shows that the globular domains of different isoforms bind to the nucleosome dyad, while the IDR at the C-terminus determines the orientation of the linker DNA^90^. H1 induces phase separation in the presence of very short DNA (~100 bp), and the H1-condensate size is invariant with DNA or polynucleosome length, likely indicating that SIPS is the main mechanism, although further investigation with longer DNA may be needed^89^. Phase separation studies in HeLa cell nuclei revealed that H1 condenses into heterochromatin and colocalizes with HP1α.

Heterochromatin protein 1 (HP1) is known to induce the formation of constitutive heterochromatin through phase separation^19,20^. A series of in vitro experiments revealed that HP1α initiates phase separation when it is phosphorylated or when it interacts with DNA, likely through a conformational change that allows interactions between dimers^19^. This study showed that phase separation via multivalent interactions between the N-terminus and hinge of HP1α is stimulated by phosphorylation. Its ortholog *Drosophila* HP1a also exhibited phase separation both in vitro and in vivo^20^. This suggests that the phase separation type of HP1α is SIPS. In contrast, another in vivo study suggested that HP1α condensates show characteristics indicating BIPS instead of SIPS^88^. The condensates did not grow in size as the bulk HP1α concentration increased. Instead, the concentration of HP1α within the condensate increased, which is expected in BIPS. HP1α interacts with the H3K9me3 of a nucleosome via its chromodomain (CD) and acts as a bridge between two nucleosomes^91^. In addition, because HP1α is a reader of H3K9me2/3, the interaction mechanism depends on epigenetic modifications. Hi-C studies have shown that epigenetically marked regions, which act as blocks on a block copolymer, are bridged by HP1α, which induces the formation of compact, heterochromatin-like structures^91^. Furthermore, HP1a has been shown to bridge two separate chromosome sites in *Drosophila*^92^. Therefore, bridging between nucleosomes provides another possibility for BIPS by HP1α. Hence, in this case, interplay between BIPS and SIPS can be used to explain the formation of condensates^92^. These results suggest that BIPS can provide a platform for the growth of HP1-nucleosome condensates by recruiting HP1α condensates that are formed by SIPS^93^.

The two polycomb repressive complexes PRC1 and PRC2 are chromatin-modifying complexes that were initially discovered to silence the Homeotic genes of *Drosophila*^94–97^. These two proteins are known to spread the H3K27me3 histone mark, PRC1 initiating the first mark and PRC2 spreading it, to regulate facultative heterochromatin. PRC1 monoubiquitylates histone H2A at Lys119, whereas PRC2 monomethylates, demethylates, and trimethylates histone H3 at Lys27^97,98^. Both PRCs have been shown to initiate phase separation in the presence of DNA^99,100^ and to interact with nucleosomes. In particular, PRC2 has multivalent DNA-binding sites^100^, and AFM images have shown that PRC2 can bridge DNA^101^. Therefore, the PRC proteins seem to use BIPS to condense chromatin.

## Physiological roles of BIPS and SIPS

BIPS and SIPS affect genome structure and function (Table 4). For example, cohesin is known for its ability to extrude DNA loops via ATP hydrolysis, thereby creating boundaries of topologically associating domains (TADs) and building up the genome structure. Chromatin contacts end abruptly between TAD borders, and interchromatin contact is preferred within each domain^101^. These loop domains are disrupted by the elimination of cohesin, and the disruption of related proteins, such as CTCF or WAPL, alters loop formation^102^. Cohesin has also been shown to form phase-separated clusters on DNA via BIPS, suggesting that cohesin condensates are also used to construct chromosome structures^40^. According to a recent study, MD simulations have shown that both DNA-loop extrusion and BIPS partitions induced by a strings and binders (SBS) model can coexist in chromatin shaping^102^. Similarly, in some bacteria, such as *Bacillus subtilis*, ParB can bridge DNA via dimerization and furthermore pack and condense DNA by phase separation at the origin of replication. However, the physiological implications of ParB condensates are still not fully understood.

**Table 4:** Examples of genomic functions related to phase separation.

Functions	DNA components	Protein components	SIPS/BIPS	Functional characteristics	References
Superenhancers	Enhancers, Promoters	TFs, Transcriptional coactivators, pol II, Cohesin, CTCF, Med1	[SIPS] Interaction between IDRs of protein components		^103^
			[BIPS] Cohesin-CTCF loops	Cohesin-CTCF loop forms a nucleation site for pol II clustering.	^105^
Silencing	Heterochromatin DNA	HP1, PRC1, PRC2	[BIPS + SIPS]		
	AT-rich DNA	H-NS	[BIPS] DNA bridging by H-NS dimers	Bridged H-NS complex can compact DNA.	^125^
	Transposons	MORC1	[BIPS] Loop-trapping by MORC1 dimers	MORC1 can silence transposons by bridging DNA and clustering.	^107^
Splicing (nuclear speckle interfacial splicing model)	[Inside speckle] Exons (enriched in specific sequence motifs that recognize serine-rich and arginine-rich (SR) protein), RNA	SR proteins	[SIPS] Interaction between IDRs of protein components and RNA	Nuclear speckle shows exclusion by differences in chemical environment.	^109^
	[Outside speckle] Introns (enriched in hnRNP sequence motifs)	hnRNPs			
	[Peripheries of speckle] Splicing site	Spliceosomes		The interface of a phase-separated droplet recruits spliceosomes, making them functional.	

BIPS and SIPS can also influence transcription. For example, superenhancers are clusters of enhancers that are believed to be formed by phase separation^103,104^. The proteins involved are bound via multivalent interactions with IDRs, while the DNA sites are bound by master transcription factors. Superenhancers have high densities of transcriptional machinery, driving robust expression of genes with prominent roles in cell identity. CTCF depletion has been shown to prevent the formation of Pol II clusters in cells, mostly at superenhancers^105^. In the same study, looping between enhancers and promoters by CTCF was suggested to influence the clustering of Pol II and other molecules by creating a structural hub for Pol II via loop extrusion. This might be an example of BIPS forming a structural hub for liquid-liquid phase separation (LLPS) to occur.

Another role of BIPS or SIPS is gene silencing. Heterochromatin formation occurs via heterochromatin packaging by HP1 or chromatin remodeling by PRC1 and PRC2. Repression by heterochromatin formation silences a wide range of genes. A more specific method of silencing can be found in H-NS, mentioned above, and in the Microrchidia (*Morc*) family of ATPase proteins. MORC proteins are critical for gene silencing and chromatin compaction in various eukaryotic systems, for example, in silencing transposons^106^. MORC1 has been shown to form clusters on DNA in vitro, where its propensity to bind to free DNA suggests a loop-trapping mechanism, and it preferentially binds to longer DNA^107^. This serves as an example of DNA compaction due to BIPS acting as a gene silencing mechanism.

The nuclear speckle interfacial splicing model is notable because it describes the role of the interface of a membraneless body. Nuclear speckles are irregularly shaped bodies in the interchromatin space that are found near gene-rich regions or active transcription sites^108^. These speckles are phase-separated membraneless bodies formed by various RNAs and RNA binding proteins, particularly pre-mRNA splicing factors. These RNA-binding proteins contain low-complexity IDRs that cause self-association and phase separation^109^. Immunofluorescence and fluorescence in situ hybridization studies have shown spliceosomes located at the periphery of the speckles^110^. The inside of the speckles is enriched with exons and SR proteins, and the outside is enriched with introns and hnRNPs, respectively^109,111,112^. The nuclear speckle interfacial splicing model is based on the fact that exonic sequences are enriched with SR motifs and that intronic sequences are enriched with hnRNP motifs. The pre-mRNA exon is positioned within the nuclear speckle, and the splice site motifs are at the periphery, allowing spliceosomes to perform their catalytic activity^110^.

## Conclusions and future perspectives

Phase separation can explain long-standing, unresolved questions in genomic organization and function, such as RNA transcription, genome structure formation, and DNA repair. However, the molecular mechanisms underlying the formation of countless chromosomal phase-separated condensates are still under debate. In this review, we introduce two potential working models behind chromosomal phase separation and describe how they are involved in chromosome function. We introduce BIPS, in which a bridged DNA loop serves as the nucleation point for phase separation. Because chromosomes contain extremely long DNA molecules, DNA topology should be considered in elucidating the underlying molecular mechanism. BIPS is different from the typical phase separation mechanism, called SIPS, which is induced by self-interaction between multivalent protein‒protein interactions. We show some examples of these two mechanisms and suggest that these mechanisms can be commonly applied to other chromosomal phase-separated condensates. Although biophysical modeling has been applied to understand these molecular mechanisms, more detailed and complex circumstances must be considered to understand how BIPS and SIPS combine to determine genome structure and function^113,114^.
